# Bioarchaeological perspective on the expansion of Transeurasian languages in Neolithic Amur River basin

**DOI:** 10.1017/ehs.2020.16

**Published:** 2020-05-14

**Authors:** Yinqiu Cui, Fan Zhang, Pengcheng Ma, Linyuan Fan, Chao Ning, Quanchao Zhang, Wei Zhang, Lixin Wang, Martine Robbeets

**Affiliations:** 1 Research Center for Chinese Frontier Archaeology, Jilin University, Changchun 130012, China; 2 School of Life Sciences, Jilin University, Changchun 130012, China; 3 Eurasia3angle, Max Planck Institute for the Science of Human History, Jena D-07745, Germany; 4 School of Archaeology, Jilin University, Changchun 130012, China; 5 Heilongjiang Provincial Institute of Cultural Relics and Archaeology, Harbin 150008, P. R. China

**Keywords:** Ancient genome, Transeurasian languages, population migration, agricultural dispersal

## Abstract

Owing to the development of sequencing technology, paleogenomics has become an important source of information on human migration and admixture, complementing findings from archaeology and linguistics. In this study, we retrieved the whole genome and Y chromosome lineage from late Neolithic Honghe individuals in the Middle Amur region in order to provide a bioarchaeological perspective on the origin and expansion of Transeurasian languages in the Amur River basin. Our genetic analysis reveals that the population of the Amur River basin has a stable and continuous genetic structure from the Mesolithic Age up to date. Integrating linguistic and archaeological evidence, we support the hypothesis that the expansion of the Transeurasian language system in the Amur River basin is related to the agricultural development and expansion of the southern Hongshan culture. The spread of agricultural technology resulted in the addition of millet cultivation to the original subsistence mode of fishing and hunting. It played a vital role in the expansion of the population of the region, which in its turn has contributed to the spread of language.

## Graphical abstract

**Figure:**
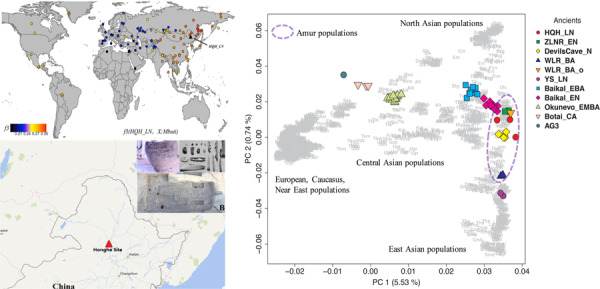

**Media summary:** Agriculture emerged in the Amur River basin in the late Neolithic. It remains unclear what caused the transition from fishing and hunting to agriculture and whether it can be associated with population migration and language spread. We retrieved genomic data from late Neolithic Honghe individuals in the Amur River basin and provided a bioarchaeological perspective on the origin and expansion of Transeurasian languages in the Amur River basin. Our genetic analysis supports the hypothesis that the expansion of the Transeurasian languages in the Amur River basin is related to agricultural development and expansion of the southern Hongshan culture. The spread of agricultural technology resulted in the addition of millet cultivation to the original subsistence mode of fishing and hunting.

## Introduction

Humans have been on the move throughout their long history, spreading their language and culture with them. Archaeologists study material remains of past human life to understand minor and major innovations in culture across time. Historical comparative linguists compare words between related languages to recover certain aspects of the unattested ancestral language and how it changed over time. In addition to material remains and languages, the human genome also retains traces of the past. With the development of sequencing technology, beginning around 2010, genomic data can be retrieved directly from ancient human remains. These data provide an independent source of information to understand the biological relationships among populations across time. Comparing ancient genomes with those of current populations can reveal something about the origins of the ancient populations, previous population movements and admixture events (Lazaridis et al., 2016; Llamas et al., 2016). Thus, a bioarchaeological perspective is expected to complement linguistic and archaeological approaches as a source of knowledge on prehistoric human populations and migrations.

The Amur River (AR) originates in the eastern foothills of Kent, Mongolia, and rises at the junction of the Selenga River and the Argun River. Passing through the northeastern part of China, it receives many tributaries, such as the Zeya, the Songhua, the Nen and the Ussuri Rivers. In Khabarovsk it ceases to define the Russian–Chinese border until it finally enters the Pacific Ocean through the Tartar Strait. The AR basin is rich in aquatic resources and includes diverse ecosystems such as forest-steppe and dry steppe landscapes in the upper reaches in the west, forest-meadows in the middle reaches and grassland in the eastern lowlands. These geographical and natural features have made the AR basin a corridor connecting the northern Asian inland with the northern Pacific coastal area.

Archaeological studies have shown that, from the Mesolithic Age, the subsistence pattern of AR populations mainly relied on fishing and hunting. The emergence of pottery in the Neolithic period was not associated with agricultural activities as the evidence of farming is quite late in comparison with pottery making (Kuzmin, 2014). The pottery found at Neolithic sites of the Nen River basin in the central Amur region, such as Houtaomuga and Honghe, is flat-bottomed and differs from the round or pointed bottoms that appeared in the Japanese archipelago, the Russian Far East and the Korean Peninsula. This points to a unique and independent cultural tradition in the AR basin even if the fact that early pottery is widely distributed among different pre-agricultural societies in East Asia, but not found among pre-agricultural societies elsewhere, may suggest that it initially spread in East Asia through early intersocietal interactions (Shelach, 2012). From the late Neolithic and early Bronze Age onwards, mammalian bones such as cattle and deer have been found in ash pits and ditches, rather than fish bones, shells and bird bones. Together with evidence of millet and grinding stones, this was taken as an indication of the transition from fishing and hunting to agriculture and animal husbandry (Wang & Sebillaud, 2019). Nevertheless, it remains unclear what caused the developments that led to the emergence of agriculture and whether it can be associated with population migration and language spread.

The AR region is home to a variety of ethnic groups speaking languages belonging to different families, such as Tungusic, Mongolic, Amuric (Nivkh), Sino-Tibetan (Mandarin) and Indo-European (Russian) languages. The general presence of Russian and Mandarin is due to a relatively recent expansion of these languages in the region. Nivkh goes back to an ancient local lineage that became isolated when the Tungusic and Mongolic languages expanded. The latter languages are thought to derive from an ancestral Transeurasian language, the descendants of which are widespread across Eurasia. A partial explanation for this wide distribution may be the adoption of agriculture by the early speakers.

In spite of controversy in the linguistic literature (Johanson, 2010), the hypothesis that Japanese, Korean, the Tungusic, the Mongolic and the Turkic languages are geneaologically related is gradually gaining acceptance (Robbeets, 2005, 2015). The term ‘Transeurasian’ used in reference to this language family replaces the classical term ‘Altaic’. The homeland where the common ancestral language was once spoken is situated in the West Liao River basin and inferred at a time depth of 6700 BP (highest posterior density interval 4330–9450 BP) (Robbeets & Bouckaert, 2018). From this location, the Transeurasian languages are thought to have spread across the Amur basin to the Southern Primorye, over the Liaodong Peninsula to Korea and Japan and over the eastern Steppe as far as Anatolia. Although agriculture has been proposed as a driving force (Robbeets, 2017), it remains unclear what caused the language spread and whether it can be associated with population migration.

Genetic studies of contemporary populations of the AR basin indicate that the genetic structure of these populations is very similar, even if they belong to different linguistic groups. Previous studies investigating the paternal Y chromosome, for instance, have found evidence for shared ancestry among Tungusic and Mongolic populations. Y-chromosomal haplogroup C-M130 is widespread over a large area encompassing both Siberia and the AR basin (Zhong et al., 2011). This haplogroup has been found in high frequencies, not only in speakers of Transeurasian languages such as Tungusic- and Mongolic-speaking populations, but also in speakers of non-related languages such as Nivkh. Until recently, research on the correlation between genetic and linguistic lineages has mainly been based on the study of modern populations living in the AR basin. However, owing to large-scale expansion, admixture and replacement of both languages and genes in relatively recent times, it is difficult to reconstruct the migration history of current ethnic and linguistic groups from the AR basin on the basis of modern data alone. Therefore, we will here use ancient DNA to shed light on the genetic structures of ancient populations in this area.

In this study, we will focus on the genetic structure of the late Neolithic Honghe population from the Middle Amur region. By analyzing the whole genome structure and Y chromosome lineage of this ancient population, we will contribute to the understanding of the Amur genetic profile in the Neolithic. We intend to map our genetic findings on those from archaeology and linguistics in order to provide a bioarchaeological perspective on the origin and expansion of Transeurasian languages in the AR basin.

## Materials and methods

### Materials

**Figure 1.**
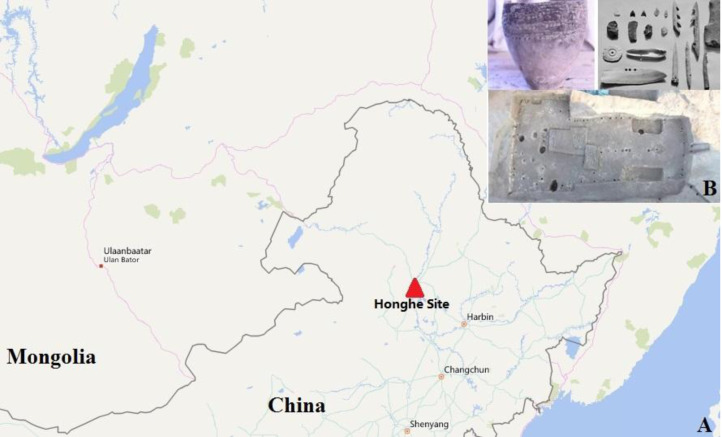
Geographic location (a) and archaeological relics (b) of the Honghe site.

The Honghe Site is situated in Honghe Village, Qiqihar City, Heilongjiang Province of NE China. The site is dated to around 4000 BP and belongs to the cultural context of the Ang'angxi culture, the ‘Fishing–Hunting Neolithic Culture’ on the middle reaches of the Nen River between 7000 and 4000 BP. The whole site is distributed in the shape of a narrow bar with a width of only 100 metres and a length of 10,000 metres (Figure 1). The unearthed artefacts from the Neolithic period contain jars, bowls, cups and stone artefacts.

The use of ancient samples in this study was approved by the Heilongjiang Provincial Institute of Cultural Relics and Archaeology and Jilin University. The teeth from four individuals were sent to our ancient DNA laboratory in Jilin University, which contains the positive-pressure clean rooms used for sample preparation, DNA extraction, PCR and library construction separately. Post-library procedures were carried out in a different building.

In order to reduce modern contamination, we collected intact molars for testing. Teeth were soaked in 5% sodium hypochlorite solution for 10 min, then washed with distilled water and ethanol. Each side of the teeth was exposed to ultraviolet (UV) light (254 nm) for 30 min. This allowed us to remove most contamination from soil and researchers. The archaeological and anthropological data of the ancient individuals is shown in Table 1.

**Table 1.** Sample information and nuclear human DNA screening of Honghe site individuals

Individual ID	Population label	Tissue	Period	Genetic sex	Endogenous DNA (%)	Mean coverage	mtDNA contamination	mtDNA haplogroup	mt mean coverage	Y-hg	Y mean coverage
**HQHM2**	HQH_LN	Upper third molar	Late Neolithic	Male	11.767	0.0425	0.008	D4c1b	52.0482	C2a	0.019
**HQHM3**	HQH_LN	Upper second molar	Late Neolithic	Male	1.621	0.0067	0.009	D4j	17.6003	—	0.003
**HQHM4**	HQH_LN	Upper third molar	Late Neolithic	Male	15.85	0.0433	0.022	D4b2a	59.4463	Q1a1a, F1907-	0.02
**HQHM5**	HQH_LN	Lower third molar	Late Neolithic	Female	7.008	0.0213	0.016	D4	20.8831		

### Sample preparation and sequence data processing

All four genomic DNAs were extracted from teeth and the double-stranded libraries of Honghe individuals (HQH_LN) were prepared following published protocols (Dabney et al., 2013; Meyer & Kircher, 2010). All libraries were sequenced on Illumina HiSeqX10 platform in a double index configuration. The raw FASTQ files were processed using EAGER v1.92.50 program (Peltzer et al., 2016). Ancient DNA data processing adapters were trimmed with Adapter Removal v2.2.0 and read lengths longer than 30 bp were mapped to the human reference genome (hs37d5) using BWA 0.7.12 (Peltzer et al., 2016). We used the ‘-n 0.01’ and ‘-l 1024’ parameters to allow for more mismatches and to disable the seeding. The PCR duplicate were removed with dedup v0.12.2 (Peltzer et al., 2016) and reads with mapping quality score smaller than 30 were filtered out using SAMtools (Li et al., 2009). To measure the authenticity of ancient DNA data, we applied three methods. First, we applied mapDamage v2.0.6 (Ginolhac, Rasmussen, Gilbert, Willerslev, & Orlando, 2011), with default parameters to determine the damage patterns typical of ancient DNA. Second, the contamination of mitochondrial sequences was estimated using Schmutzi (Renaud, Slon, Duggan, & Kelso, 2015). Third, to assess the contamination of the nuclear genome in males, we estimated the X chromosome polymorphism rate on male samples with at least 200 SNPs covered on Y chromosome using ANGSD v0.910 (Korneliussen, Albrechtsen, & Nielsen, 2014). To minimize the impact of the post-mortem DNA damage, the first and last 10 bases of each read were clipped based on the DNA misincorporation pattern of each library using the trimBam module in bamUtil version 1.0.13 (Jun, Wing, Abecasis, & Kang, 2015). We used SAMtools mpileup with parameters -q 30 and -Q 30 to generate a pileup file containing only sites overlapping with published 1240k panel (Fu et al., 2013; Mathieson et al., 2015) and subsequently we randomly called the genotype for each library by a pseudo-diploid method and implemented it using pileupCaller (https://github.com/stschiff/sequenceTools). Finally, we restricted our analysis to individuals with at least 15,000 target SNPs, and the HQHM3 individuals were excluded from the main analysis because of low coverage (6843 covered SNPs).

### Sex determination and kinship analysis

We assessed the biological sex of HQH_LN individuals by computing the ratio of X chromosome-derived shotgun sequencing data to the autosomal coverage. To determine the genetic kinship between ancient individuals, we applied the methods of pairwise-mismatch rate analysis (Kennett et al., 2017).

### Analysis of uniparental genetic markers

We used GATK (DePristo et al., 2011) Unified Genotyper to call variant sites for mitochondrial genomes. We then filtered off heterogenous and low-quality sites, and compared the remainder with a list of variants reported in phylotree-Build 17 (http://www.phylotree.org) to determine mtDNA haplogroups using self-made script. The variants of terminal haplogroup were double-checked using IGV software (Robinson, Thorvaldsdottir, Wenger, Zehir, & Mesirov, 2017).

Based on our massive modern population data, a BED file which contained callable regions of Y genomes was produced, and then we used it in bcftools to call variants. After filtering off heterogenous and low-quality sites, we compared the list of variants with SNPs based on the ISOGG 2018 tree (http://www.isogg.org/tree) together with our own most updated Y-tree to determine Y haplogroups using a self-made script. The variants of terminal haplogroup or those in doubt were double-checked using IGV software (Robinson et al., 2017).

### Genomic analyses

We merged our new data with published data of modern and ancient individuals (Allentoft et al., 2015; Damgaard et al., 2018; Jun et al., 2015; Lazaridis et al., 2016; Mathieson et al., 2015). The final dataset covers 593,124 autosomal single nucleotide polymorphisms (SNPs) in the Affymetrix Axiom Genome-wide Human Origins (HumanOrigins) array platform (Patterson et al., 2012). All population genetic analysis in this study were based on this dataset. We carried out principal component analysis (PCA) using the Smartpca in the EIGENSOFT 6.1.4 package (Gurinovich et al., 2019). We used default parameters and added two additional options (lsqproject:YES and numoutlieriter:0) to project the ancient individuals onto the first two variations (PC1 and PC2) of present-day populations. We used two datasets for the projection: the first based on all present-day Eurasians genotyped on the Affymetrix Human Origins array and the second only based on present-day East Eurasians. We computed *f*3 and *f*4 statistics using the qp3pop and qpDstat packages in ADMIXTOOLS, respectively (Patterson et al., 2012). We used the ‘inbreed: YES’ parameter to calculate admixture *f*3 statistics as a test for admixture with HQH_LN as a target group and all other genomes as sources. For computing the *f*4 statistic, we used the ‘*f*4mode: YES’ parameter.

## Results

Strict procedures were applied to minimize modern DNA contamination. We confirmed the authenticity of our results with a number of different observations: (a) the negative extraction and amplification controls were free of contamination; (b) the nucleotide misincorporation patterns characteristic of ancient DNA at the 3′- and 5′- ends of the DNA sequences were observed (Figure S1); (c) the sequences show very low contamination estimates for mtDNA (1–2%; Table 1); and (d) blank controls were carried along during every step of library preparation. Amplified libraries of the blank controls were quantified with Qubit® fluorometric quantitation and did not exhibit any amplification, indicating that exogenous contamination was minimized in the analysis.

We have classified the Y chromosome of two male samples in Honghe Village, Ang'angxi Culture, as shown in Table 1, HQHM2 belongs to C2a. Owing to the low coverage of the data, only very limited Y-SNPs were covered and it is hard to further subdivide them into downstream sub-branches. The haplogroup C2a is distributed at high frequency in the modern populations of the AR basin and Siberia, such as the Negidal, Udehe, Oroqen, Hezhen, Dagur, Khalkha Mongolians and Kazakh, with a specially high diversity in the AR population (Wei et al., 2017). Another sample HQHM4 belongs to Q1a1a and shows the characteristics of early branches. Q1a1a is mainly distributed in central and southern East Asian populations such as Han, Naxi and Vietnam in the modern population, but rare in Siberia and Central Asia (Sun et al., 2019).

**Figure 2.**
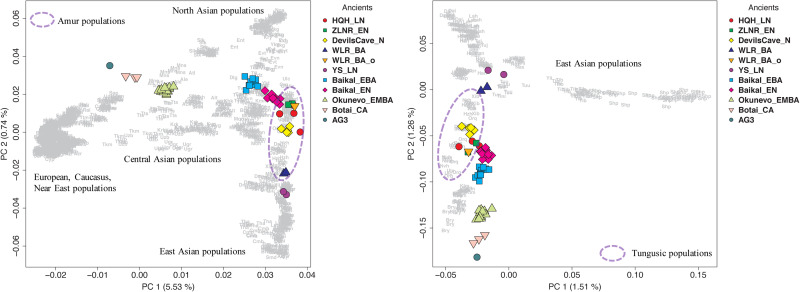
Principal component analysis on all present-day Eurasian populations (a) and only modern Asians (b), with ancient samples projected.

**Figure 3.**
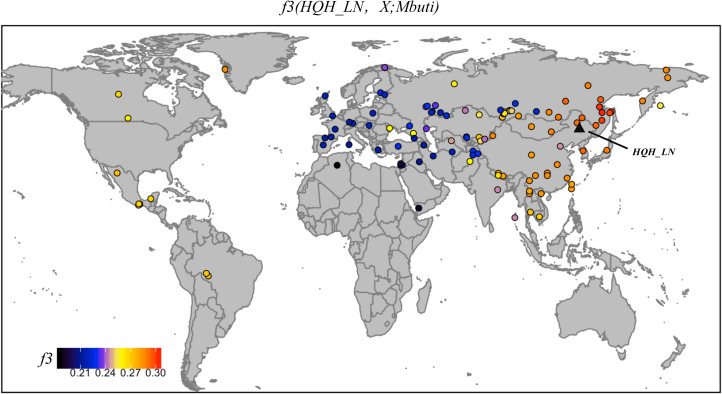
Genomic affinity of present-day human population or ancient individual to Honghe quantified by the outgroup f3-statistics of the form f3.

To understand the genetic profile of the late Neolithic Honghe individuals, we provided a PCA of present-day Eurasian populations and projected Honghe individuals and other published ancient genomes against the background of the modern populations. Whereas PC1 separates eastern and western Eurasian populations, PC2 differentiates eastern Eurasian populations along a North–South cline with northernmost Siberian Nganasans occupying one end and the Ami and Atayal from Taiwan the other. The late Neolithic Honghe population approximately occupies the intermediate position of the North–South cline and forms a tight cluster with modern Tungusic-speaking populations from the AR basin (Figure 2). Similarly, the Neolithic Devil's Cave population (Devil'sCave_N) (Siska et al., 2017) and the Neolithic Zhalainuoer individual (ZLNR_EN) from Hulunbuir, Northeast China (Ning et al.) also fall within this region. The PCA results demonstrate that the HQH_LN has strong affinities with Devil'sCave_N, ZLNR_EN and modern Tungusic speakers. To confirm the genomic relationship between ancient and modern populations from the AR basin, we further computed the outgroup f3-statistic of the form f3(HQH_LN, X; Mbuti) (Figure 3), which measures the amounts of shared genetic drift between HQH_LN and X population. Consistent with the observations from PCA, outgroup f3-statistic results confirmed that the HQH_LN people share substantial drift with present-day Tungusic populations from the AR basin, such as the Negidal, Evenki from the Far East and Ulchi, as well as with Devil'sCave_N and ZLNR_EN. As such, our results suggest a high degree of genetic continuity in the AR basin from the Neolithic to modern times. In an unpublished study, we further found a high degree of genetic similarity between the Honghe and the Houtaomuga groups, who also lived in this region between 12,000 and 2300 years ago. This suggests population stability over at least 10 millennia.

**Figure 4.**
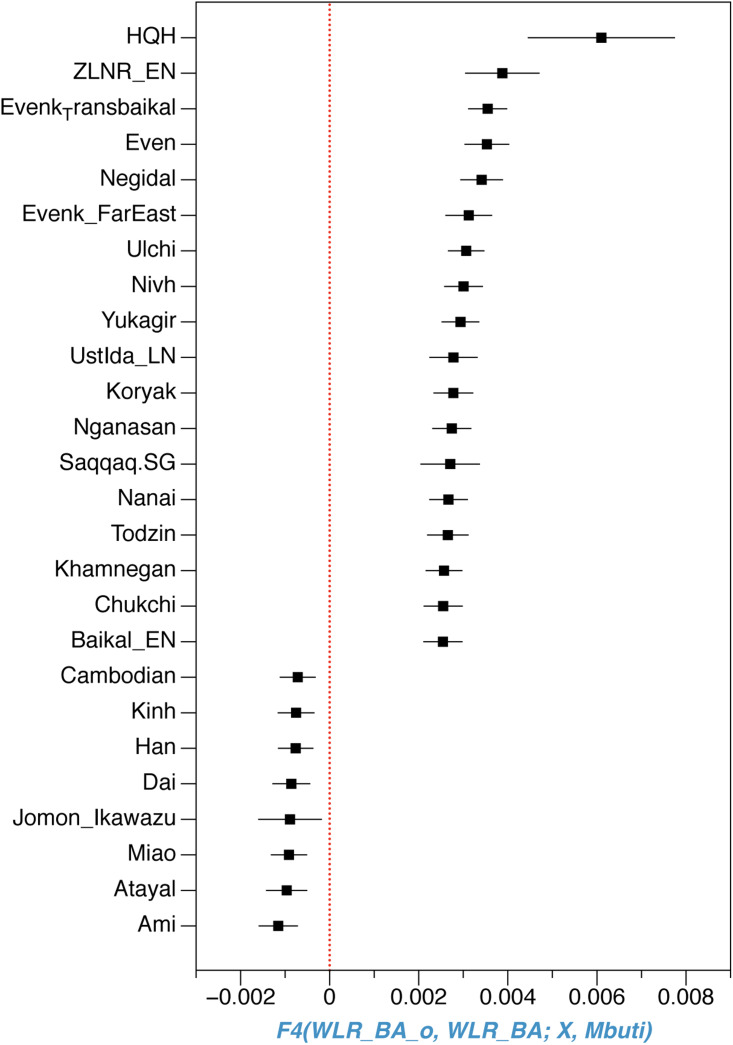
The shared genetic drift between AR basin population and an outlier individual (WLR-BA_o) from Bronze Age population in WLR region highlighted by f4-statistics of the form (WLR_BA_o, WLR_BA; X, Mbuti)

In the PCA plot, we observed noticeable genetic differences between HQH_LN with the Upper Xiajiadian culture populations from the West Liao River region (WLR_BA) and late Neolithic Yellow River populations (LS_LN) from the Central Plain of China (Ning et al., submitted). To further clarify the relationship among these ancient groups and other present-day Asians, we restricted the dataset and just carried out a PCA of all modern Asians (Figure 2b). We found that HQH_LN overlaps with present-day Tungusic-speakers, such as Oroqen, Ulchi and WLR_BA and LS_LN populations, fall within the range of modern populations from Inner China who live in the same geographic region. Based on the PCA and *f*3-statistics analyses, we concluded that the HQH_LN has less genetic affinity with WLR_BA and LS_LN. In addition, we observed that HQH_LN, as other populations in the AR basin, had a close genetic relationship with an outlier individual (WLR-BA_o) in the Upper Xiajiadian culture populations from WLR region (Figure 4).

## Discussion

Our genetic analysis reveals that the population of the AR basin has a stable and continuous genetic structure from the Mesolithic Age up to date. As shown on the PCA plot, ancient AR basin individuals, belonging to different periods and different sites, form a tight cluster with contemporary populations in this region (Figure 2). This is confirmed by the *f*3 analysis. Integrating observations from archaeology, our genetic results suggest that the Amur genetic profile is reflected in the early fishers and hunters in the region and continues in the ensuing millet farmers. As the AR basin is rich in aquatic systems and wildlife resources, the people here developed a subsistence pattern that heavily relies on fishing and hunting. The emergence of agriculture in this area came relatively late, notably with the cultivation of millet in the late Neolithic. In Northeast China, broomcorn and foxtail millets were first cultivated by 7600 years ago south of the AR basin, within the context of the early Neolithic Xinglongwa culture (8200─7500 BP) in the West Liao River basin (Stevens & Fuller, 2017; Zhao, 2011). However, millet did not spread beyond the West Liao River basin until the Middle–Late Hongshan (6000–5000 BP) period. Broomcorn and foxtail millets found at the Wangjiacun site suggest that millet agriculture reached the Liaodong region by 5500–5300 BP (Ma, Wu, Wang, Zhang, & Jin, 2015). At approximately the same time millet dispersed westward to Central Asia. Agricultural tools were discovered at Tengjiagang (6000–5000 BP) in Heilongjiang, as well as at the Yabuli and Yinggeling sites in eastern Jilin and Heilongjiang (Tianxiang, Guozhen, & Hu, 1981; Yantie, 1988). In the Russian Far East, the oldest millet finds are from Krounovka 1 and go back to 5620–5370 BP (Kuzmin, 2013; Sergusheva, 2008). It is, therefore, probable that millet agriculture had spread to eastern Jilin and Heilongjiang and from there to the Russian Far East no later than 5500 BP (Li et al., 2009) and we can assume continuing contacts between the WLR basin and the AR basin between 6000 and 4000 BP (H. Li et al., 2011).

The analysis result of f4 and PCA showed that the people in the AR basin kept contact with the surrounding population while maintaining their own stability, but the migration is in an outward one-way direction as we observed from the genetic data. As shown in the PCA plot, the ancient individuals from Honghe site in the AR basin are quite close to the Devil's Gate individuals from the Russia Far east and almost overlap with the main cluster of Tungusic-speaking populations, which indicates a high genetic affinity between individuals from the Honghe and Devil's gate (Siska et al., 2017) and suggests that the Amur-related ancestry extended across a large geographic area, from the Russian Far East to the Liao River region. However, the southern neolithic agricultural populations from the West Liao River (Middle–Late Hongshan culture) and the Central Plains (Longshan Culture) are separate from Honghe peoples as they are located along the north–south axis on the right side of the PCA. As the West Liao River genome from the Middle–Late Hongshan culture is drawn less towards that of the Central Plain than the West Liao River genome from the Bronze Age, there is reason to assume that the West Liao River genome over time became less Amur-like (Ning et al.). That is to say, if the spread of millet agriculture from the West Liao River region to the AR Region was driven by population migration, we would not be able to perceive this from the genome because it would have resulted in an admixture between two similar Amur genomes. Thus, the situation may be similar to that of Anatolian farmers entering Europe (Lazaridis et al., 2016), except that it is not visible in the genome. In contrast, the spread of dairy products from the western Eurasian steppe to the Mongolian plateau was driven by cultural diffusion and not accompanied by large-scale infusion of genetic components from West steppe herders (Jeong et al., 2018).

Among the languages spoken in the AR basin, we find Transeurasian languages as well as non-Transeurasian languages. The Transeurasian languages include Tungusic languages, such as Even, Evenki, Oroqen, Solon, Negidal, Nanai, Udehe, Oroch, Olcha, Orok and Manchu, and Mongolic languages, such Khalkha and Buryat. The earliest AR populations mentioned in Chinese historical records whose languages are thought to have comprised Transeurasian elements are the presumably Mongolic Donghu (ca. 300–150 BC) and Xianbei (130–180 AD) tribes of Western Manchuria, the presumably Japanic Puyo (Fuyu) in the Sungari basin and the presumably Tungusic Sushen (ca. 300 BC), later named ‘Yilou’, of Eastern Manchuria (Robbeets, 2015). Apart from Russian and Chinese who came to dominate the Amur region in historical times, a non-Transeurasian language native to the Amur region is Nivkh, usually considered as a marginal pocket of lineages that became isolated before Transeurasian language spreads.

From our study it appears that there is a long-term genetic stability in the Amur region, whereby the contemporary speakers of the Tungusic, Mongolic and Nivkh in the region are genetically continuous with the ancient Amur populations. This indicates that the contemporary populations may have a common ancestral population with an Amur genetic profile. Thus, when the Transeurasian languages spread and local people of non-Transeurasian languages abandoned their own language and shifted to Transeurasian, this resulted in admixture between different populations with Amur genome. C2a is the core Y-chromosome haplogroup in the extant populations from the AR basin. It is represented in speakers of Tungusic languages such as Oroqen, Manchu, Xibe and Hezhe as well as in speakers of Mongolic languages such as Khalkha Mongolian and Buryat (Wei et al., 2017). So far, published ancient samples belonging to C2a come from the early Neolithic Age Devil's gate site from Russia Far east (7500 BP) (Siska et al., 2017), the early Neolithic Age Shamanka site from Baikal lake region (7500 BP) (Damgaard et al., 2018) and the Medieval Age Saka site from Western Tianshan (500–900 BP) (Damgaard et al., 2018), most of which are distributed in the vast area from the AR basin to the Altai–Tianshan Mountains. In our study, one Honghe male belonged to C2a. It is remarkable that our ancient samples are far apart from each other and belong to different subhaplogroups according to the accumulating Y-SNPs, which reflects the high diversity of C2a in the region. Nevertheless, they are closely clustered in the PCA plot based on genomic data. It can be inferred that there is a close exchange between the ancient populations in the AR basin throughout the Neolithic age or even later era.

To sum up, we hypothesize that the expansion of the Transeurasian language system in the AR basin is related to the agricultural development and expansion of the southern Hongshan culture. Although there is no visible sign of immersion with genetic components, population admixture of different Amur-like profiles was probably involved. The spread of agricultural technology has enabled the cultivation of millet to be added to the original subsistence mode of fishing and hunting. It has played a vital role in the expansion of the population of the region, which in its turn has contributed to the spread of language.

## Data Availability

We obtained permission from Jilin University to conduct genomic analysis of ancient samples from the Honghe Site. Please email cuiyq@jlu.edu.cn for the data set used in this study.

## Supplementary material

For supplementary material accompanying this paper visit http://dx.doi.org/10.1017/ehs.2020.16. click here to view supplementary material
