# Supplementary figures and images for: Bioarchaeological perspective on the expansion of Transeurasian languages in Neolithic Amur River basin
Source: Evol Hum Sci. 2020 May 14;2:e15. doi: 10.1017/ehs.2020.16 (PMC10427477; doi:10.1017/ehs.2020.16)

## A. HQHM2

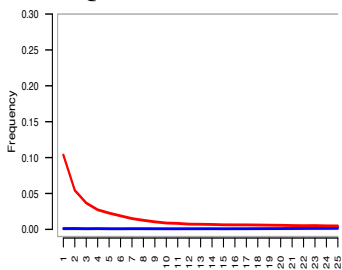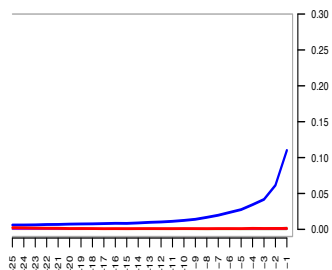

## B. HQHM3

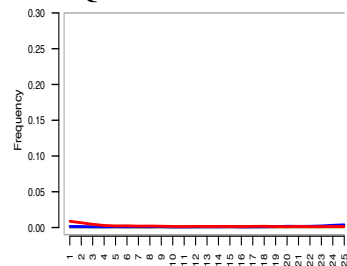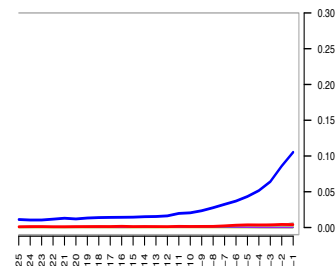

## C. HQHM4

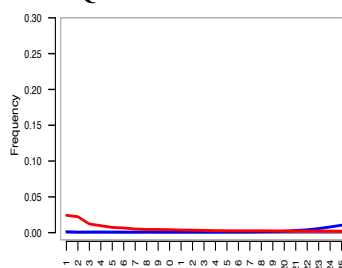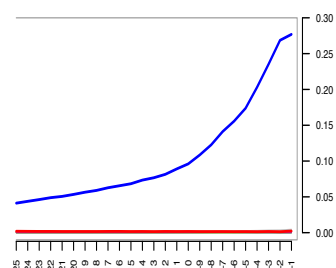

## D. HQHM5

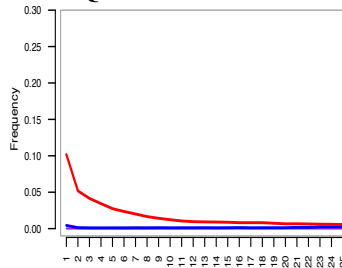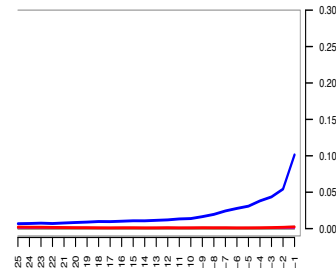

Supplement: Supplementary file 1 [file S2513843X2000016Xsup001.zip › S2513843X2000016Xsup001/FigS1. DNA damage plots for HQH_LN samples.pdf]
